# Cost-effectiveness analysis of genetic diagnostic strategies for Lynch syndrome in Italy

**DOI:** 10.1371/journal.pone.0235038

**Published:** 2020-07-01

**Authors:** Roberta Pastorino, Michele Basile, Alessia Tognetto, Marco Di Marco, Adriano Grossi, Emanuela Lucci-Cordisco, Franco Scaldaferri, Andrea De Censi, Antonio Federici, Paolo Villari, Maurizio Genuardi, Walter Ricciardi, Stefania Boccia

**Affiliations:** 1 Department of Woman and Child Health and Public Health—Public Health Area, Fondazione Policlinico Universitario A. Gemelli IRCCS, Roma, Italia; 2 Università Cattolica del Sacro Cuore, Alta Scuola di Economia e Management dei Sistemi Sanitari (ALTEMS), Roma, Italia; 3 Section of Hygiene, University Department of Life Sciences and Public Health, Università Cattolica del Sacro Cuore, Roma, Italia; 4 Department of Public Health and Infectious Diseases, Sapienza University of Rome, Rome, Italy; 5 Department of Laboratory and Infectious Sciences, Medical Genetics Unit, Fondazione Policlinico Universitario A. Gemelli IRCCS, Rome, Italy; 6 Dipartimento di Scienze della Vita e di Sanità Pubblica, Sezione di Medicina Genomica, Università Cattolica del Sacro Cuore, Rome, Italy; 7 UOC Medicina Interna, Gastroenterologia e Malattie del Fegato, Fondazione Policlinico Universitario A. Gemelli IRCCS, Rome, Italy; 8 Medical Oncology Unit, Galliera Hospital, Genova, Italy; 9 Direction of Prevention, Ministry of Health, Rome, Italy; CNR, ITALY

## Abstract

Lynch syndrome (LS) is an autosomal dominant condition caused by pathogenic variants in mismatch repair (MMR) genes that predispose individuals to different malignancies, such as colorectal cancer (CRC) and endometrial cancer. Current guidelines recommended testing for LS in individuals with newly diagnosed CRC to reduce cancer morbidity and mortality in relatives. Economic evaluations in support of such approach, however, are not available in Italy. We developed a decision-analytic model to analyze the cost-effectiveness of LS screening from the perspective of the Italian National Health System. Three testing strategies: the sequencing of all MMR genes without prior tumor analysis (Strategy 1), a sequential IHC and MS-MLPA analysis (Strategy 2), and an age-targeted strategy with a revised Bethesda criteria assessment before IHC and methylation-specific MLPA for patients ≥ than 70 years old (Strategy 3) were analyzed and compared to the “no testing” strategy. Quality Adjusted Life Years (QALYs) in relatives after colonoscopy, aspirin prophylaxis and an intensive gynecological surveillance were estimated through a Markov model. Assuming a CRC incidence rate of 0.09% and a share of patients affected by LS equal to 2.81%, the number of detected pathogenic variants among CRC cases ranges, in a given year, between 910 and 1167 depending on the testing strategy employed. The testing strategies investigated, provided one-time to the entire eligible population (CRC patients), were associated with an overall cost ranging between €1,753,059.93-€10,388,000.00. The incremental cost-effectiveness ratios of the Markov model ranged from €941.24 /QALY to €1,681.93 /QALY, thus supporting that “universal testing” versus “no testing” is cost-effective, but not necessarily in comparison with age-targeted strategies. This is the first economic evaluation on different testing strategies for LS in Italy. The results might support the introduction of cost-effective recommendations for LS screening in Italy.

## Introduction

Colorectal cancer (CRC) is the third most common malignancy and the third leading cause of cancer death worldwide, accounting for approximately 1,800,000 new cases and 881,000 deaths in 2018 [1, 2]. In Italy, CRC is the second most commonly diagnosed form of cancer in the population, after breast cancer, with more than 49,000 new cases reported in 2019, and is the second leading cause of cancer death with 20,000 deaths in 2016 [3].

Lynch syndrome (LS), previously known as hereditary non polyposis colorectal cancer, is an autosomal dominant disorder caused by a pathogenic sequence variant in one of four DNA mismatch repair (MMR) genes (*MLH1*, *MSH2*, *MSH6*, and *PMS2*). It accounts for about 3% of newly diagnosed CRC cases [4, 5]. When applying this frequency to the Italian prevalent cases of CRC, it can be estimated that in Italy almost 6,000 cases have LS.

LS has an estimated prevalence in the general population of 1/440 [6], and it is characterized by an increased risk for CRC (life-time risk: 30% to 70%) and extracolonic cancers, including endometrial (life-time risk: 30–51%) and ovarian cancer (life-time risk: 4–15%) [7, 8]. Tumors arising in LS usually show loss of expression of at least one of the MMR proteins. However, approximately 10% of sporadic CRCs also show loss of MLH1 due to methylation of the *MLH1* promoter.

Current screening recommendations for LS patients include colonoscopy every 1–2 years beginning at age 20–25 years, as well as annual transvaginal ultrasound of the uterus and ovaries, and endometrial sampling [7, 9, 10], although the effectiveness of gynecological surveillance is not established. Furthermore, prophylactic surgery is suggested as an option to reduce the risk of gynecological cancers for women with LS [11]. In addition, a regular, long-term aspirin intake has been proposed as an effective way to reduce incidence and mortality due to CRC, and the highest impact of chemopreventive strategies is expected in patients with an established diagnosis of a hereditary predisposition syndrome, such as LS [12].

As LS is associated with an increased risk of cancer, it is important to identify carriers of MMR gene defects as early as possible using appropriate diagnostic procedures.

Traditionally, risk assessment for LS was performed using clinical criteria such as the Amsterdam Criteria or the Bethesda Guidelines [13, 14]. In 2009, the Evaluation of Genomic Applications in Practice and Prevention (EGAPP) working group and, subsequently, in 2017, the National Institute for Health and Care Excellence (NICE) recommended that all CRCs be offered screening without considering clinical or histological features for LS (“universal” screening) using either immunohistochemistry (IHC) or microsatellite instability (MSI) molecular testing [15, 16]. A positive screening test is followed by genetic counseling and DNA test for MMR alterations to establish LS diagnosis.

We reported a large heterogeneity of clinical paths to detect LS patients across Europe [17]. In Italy, although international guidelines favour the universal screening approach [15, 16], there is no organized screening pathway in place aimed to identifying individuals with LS [18]. In order to understand the management practice in Italy, we performed a series of semi-structured interviews with Italian health professionals (i.e.: gastroenterologists, oncologists, geneticists, surgeons) [19]. Moreover, the published national plan for innovation of the health system based on omics sciences suggests that regional governments define a homogenous clinical path for the detection of LS patients [20, 21].

Although there is a rise in economic evaluations concerning LS screening [22, 23], no study has ever been performed in the Italian setting. Previous cost-effectiveness analyses have shown that offering genetic testing to CRC cases and increased prevention to healthy relatives may be cost-effective. However, the current practice of LS screening remains heterogeneous, and the economic impact may vary across and within countries [24].

Our study aims to assess the cost-effectiveness of different diagnostic strategies for LS from the perspective of the Italian National Health Service with the ultimate goal of supporting the implementation of a national screening strategy. Furthermore, we aimed to identify key variables that could modify the cost-effectiveness of LS screening and that should be considered by Italian decision-makers.

## Material and methods

### Model general structure

We developed a decision analytical model to estimate the quality-adjusted life-year gained (QALYs) and costs financed by the Italian National Health Service for the provision of tests aimed at the identification of LS individuals and the management of their family members. Similar to other studies [24–26], the present analysis is based on the combined use of decision trees and Markov models structured on the natural history of the disease. Individuals with newly diagnosed CRC entered the decision tree allowing the evaluation of various diagnostic strategies differing both in terms of the number of detected pathogenic variants and related costs. Should a constitutional defect be identified, first-degree relatives (FDRs) were offered predictive germline testing for the family specific alteration (cascade screening). Markov models were used to assess the natural history of the disease among undetected FDRs who inherited the LS defect as well as among FDRs who had LS detected and thus can benefit from increased surveillance and chemopreventive strategies.

The model considers a CRC incidence rate equal to 0.09% for the determination of the eligible population (newly diagnosed CRC individuals) [3].

#### Diagnostic strategies

**Fig 1:**
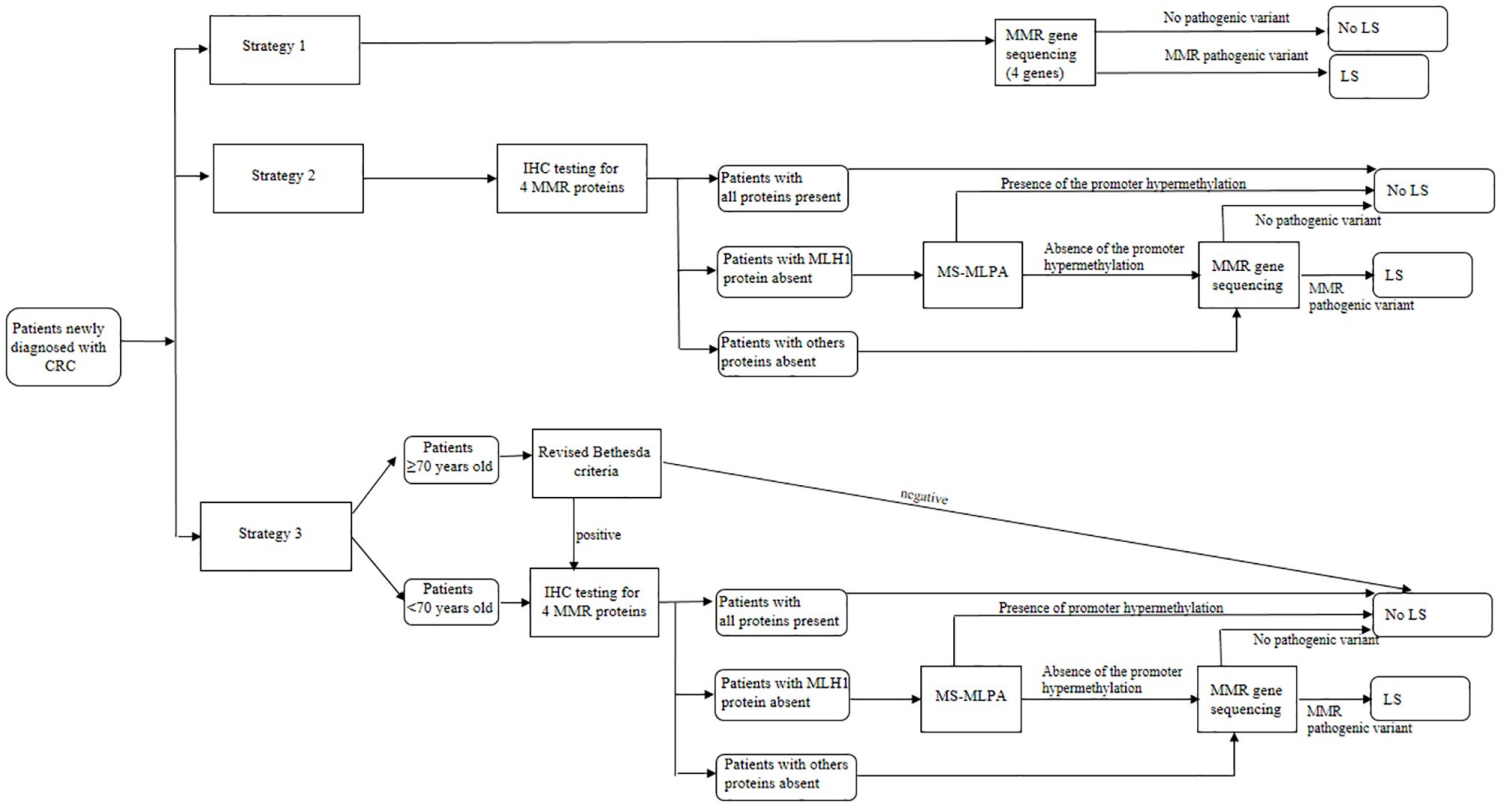
Flowchart of the three diagnostic strategies to identify LS according to Italian expert opinions [19] and [23].

The diagnostic strategies were selected taking into account Italian expert opinions [19] and the international state of the art [10, 23], and are reported in Fig 1.

Strategy 1 assumes sequencing of all MMR genes without prior tumor analysis (Next Generation Sequencing). Strategy 2 begins with offering IHC testing to all newly diagnosed patients with CRC using antibodies directed to the MMR proteins produced by the four MMR genes (*MLH1*, *MSH2*, *MSH6*, and *PMS2*). An absent stain for a protein indicates the likely presence of a pathogenic variant in the associated gene. Individuals with absent MLH1 protein staining are tested for methylation-specific MLPA (MS-MLPA) [27]. MMR sequencing of the gene coding for the absent protein is then performed in cases with absent MSH2 and/or MSH6, or PMS2 protein staining, or absent MLH1 with normal methylation pattern of the promoter.

Strategy 3 is similar to strategy 2, except that the previous procedures are offered to patients with an age at diagnosis of CRC < than 70 years old, as well as to patients diagnosed ≥70 years only if the revised Bethesda criteria are fulfilled [14].

If a causative alteration is detected in a CRC affected proband, DNA testing targeted for this variant can be offered to FDRs. FDRs who inherited the family specific variant (LS mutation carrier) are offered targeted prevention programs.

#### Markov model

**Fig 2:**
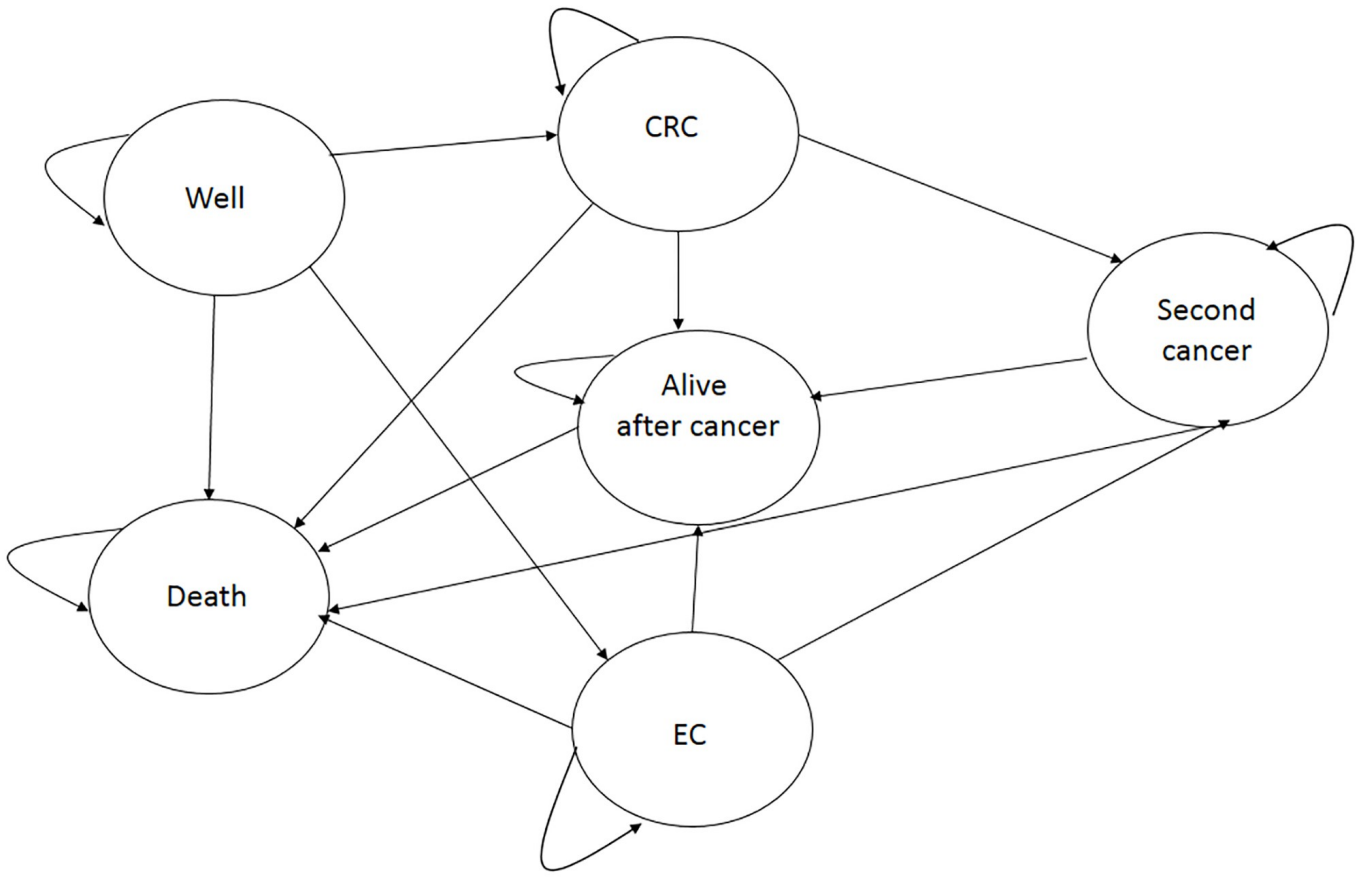
Markov model.

Tested and untested LS mutation carriers led to different Markov models, each one characterized by specific transition probabilities, 1-year cycles and a lifetime horizon reflecting the natural history of each scenario. Each Markov model consists of the following states: well, CRC, endometrial cancer, second cancer, alive after cancer, and death (Fig 2).

A FDR enters the model in the state “well” and he/she may either remain in this state or experience CRC or EC. Once CRC or EC is diagnosed, FDR may progress to the development of a second cancer. For both initial and second cancer, FDRs progress to the state “alive after cancer” if they have survived the cancer for more than 5 years.

We modeled varying levels of cancer stages for both CRC and EC as survival is dependent on cancer stage at detection, and the stage distribution is more favorable in individuals with intensified surveillance [28–31].

Following the suggestions of the Italian experts interviewed [19], the management program for LS detected FDRs (in the state “well”) included colonoscopy every 2 years beginning at age 25 and aspirin prophylaxis (100 mg/die). For women, it also included annual gynecologic visit with endometrial sampling (starting at age 35), and prophylactic surgery at age 45.

Untested FDRs who inherited LS were assumed to follow the normal surveillance of CRC patients’ relatives consisting in a colonoscopy every 5 years after 45 years.

Analyses were performed using Microsoft Excel 2013. The model considers a discount rate of 3.5% for both costs and benefits. Since an official cost-effectiveness threshold has not been defined in Italy, reference was made to economic evaluations already performed in Italy and currently available in the scientific literature where the threshold considered to assess the economic feasibility of new therapeutic strategies was equal to €30,000/QALY [32].

### Parameters

S1 Table in S1 File describes the input parameters obtained from the existing literature in the field and the semi-structured interviews [19].

#### Diagnostic strategies quality

For laboratory test sensitivity and specificity (IHC and MS-MLPA) and clinical criteria, we used estimates from Palomaki et al. [33] and Moreira et al. [34]. Predictive testing in FDR was assumed to have a sensitivity and specificity equal to 100%.

#### FDR information

Data regarding the number of FDRs per patient, the probability that relatives has LS if the patient has LS, and the mean age on entry were obtained from Severin [24] and Snowsill [35].

Uptake of genetic testing was assumed to be 84.8% for patients with cancer and 38.9% for FDRs [36, 37].

Adherence to regular colonoscopy among carrier relatives, who were positive upon genetic testing, was assumed to be 80%, and patients who adhere to colonoscopic prevention were also assumed to comply with aspirin prevention [24, 35]. The acceptance of prophylactic surgery from women with LS was assumed to be 19% [11].

#### CRC and endometrial cancer epidemiology

For the risk of CRC and endometrial cancer for healthy carriers, the values in Snowsill et al. [35] were used, where the cumulative risk of CRC up to 70 years of age is 38% for males and 31% for females. These estimates were converted into 1-year probabilities. Colonoscopic surveillance in family members was assumed to improve the distribution of such individuals among CRC stages (i.e. individuals undergoing more frequent colonoscopies are more likely to have CRC diagnosed at earlier stages). In fact, the CRC stage distribution for LS tested was different (69.4% stage 1, 25.0% stage 2, 5.6% stage 3, 0.04% stage 4) versus individuals not receiving LS surveillance (26.7% stage 1, 53.3% stage 2, 13.3% stage 3, 6.7% stage 4) [38].

The cumulative risk of EC was assumed to be 35% for women with LS [39] and mortality from EC to be dependent on time since diagnosis and independent of LS status [35].

#### Costs

The analysis investigated the direct expenses associated with the diagnostic strategies assessed, the standard prevention protocols, the enhanced preventive interventions for LS, and the general management of the disease (S2 Table in S1 File).

The costs of pharmacological treatments were estimated according to AIFA, the Italian regulatory agency for the introduction of new drugs in the National Service, and its transparency price lists for non-hospital drugs (class A). The costs of outpatient services and of surgical procedures were determined using Italian fees for specialist outpatient services [40, 41].

#### Utility

Data regarding the utilities were taken from [42, 43]. The utility of the state “alive after cancer” was assumed 0.95.

### Deterministic and probabilistic sensitivity analysis

The robustness of the results was assessed by performing both a deterministic and a probabilistic sensitivity analysis. In detail, we performed a deterministic one-way sensitivity analysis to assess the parameters whose variation deeply impacts the results obtained in the base-case (deterministic) scenario by making each parameter vary at a time. In the probabilistic analysis, the theoretical distributions of the input parameters were similar to those used in previous economic evaluations [24–26]. A total of 1000 iterations were conducted and, in order to account for the uncertainty of the input parameters, for each iteration, parameter values were randomly generated from the aforementioned distributions.

Furthermore, considering that aspirin prophylaxis is not recommended homogeneously by the international guidelines [10], we performed a scenario analysis to evaluate the cost-effectiveness trend if aspirin prophylaxis was not considered for cancer prevention.

## Results

Assuming a CRC incidence rate of 0.09% [3], and a share of patients affected by LS equal to 2.81% [5], the model identified 1,167 newly LS diagnosed cases. Table 1 shows the number of LS cases in patients newly diagnosed with CRC, the costs of the three screening strategies in CRC patients, and the costs of targeted DNA testing in the FDRs.

**Table 1:** Number of LS cases in patients newly diagnosed with CRC, costs of the screening strategies in the CRC cases, and costs of targeted DNA testing in FDRs.

Strategy	LS cases	Cost of screening in CRC cases (€)	Cost of targeted DNA testing in FDRs (€)
**No Screening**	0	-	
**Strategy 3**	910	1,753,059.93	87,257.29
**Strategy 2**	980	2,714,094.85	93,966.50
**Strategy 1**	1,167	10,388,000.00	111,886.34

Strategy 1: Next Generation Sequencing; Strategy 2: IHC, MS-MLPA, sequencing; Strategy 3: Revised Bethesda, IHC, MS-MLPA, sequencing.

Strategy 1 was the most sensitive strategy in identifying LS CRC patients (N = 1,167), followed by Strategy 2 (N = 980) and Strategy 3 (N = 910). However, Strategy 1 was also the most expensive, followed by Strategy 2 and 3. Strategy 3 has the lowest cost - €1,925.19—per LS case detected.

Table 2 shows costs, QALYs, and the incremental cost-effectiveness ratios (ICERs) as compared to “No screening” and the next most cost-effective among the remaining strategies.

**Table 2:** Cost-effectiveness analysis results based on ICER among different strategies.

Strategy	Costs (€)	QALYs	Incremental costs per QALY gained (relative to No screening) (€)	Incremental costs per QALY gained (relative to the strategy in the previous line) (€)
**No Screening**	1,505,199.28	3,369.65		
**Strategy 3**	8,407,988.32	10,703.39	941.24	941.24
**Strategy 2**	9,880,720.30	11,526.37	1,026.83	1,789.51
**Strategy 1**	18,921,334.15	13,724.50	1,681.93	4,112.86

The ICERs relative to the “No Screening” strategy ranged from €941.24/QALY in Strategy 3 to €1.681,93/QALY in Strategy 1, whereas the ICERs relative to the next most cost-effective strategy ranged from €941.24/QALY in Strategy 3 to €4.112,86 /QALY in Strategy 1. The analysis also included the realization of a scenario where the cost drivers associated to the preventive monitoring (gynecologic visit, transvaginal ultrasonography, biopsy and prophylactic abdominal hysterectomy) where excluded. The results of such scenario are reported in Table 3.

**Table 3:** Cost-effectiveness analysis results based on ICER among different strategies (no preventive monitoring).

Strategy	Costs (€)	QALYs	Incremental costs per QALY gained (relative to No screening) (€)	Incremental costs per QALY gained (relative to the strategy in the previous line) (€)
No Screening	1,441,792.53	3,369.65		
Strategy 3	7,350,880.05	10,703.39	805.74	805.74
Strategy 2	8,742,331.04	11,526.37	895.03	1,690.74
Strategy 1	17,565,848.77	13,724.50	1,557.15	4,014.10

Strategy 1: Next Generation Sequencing; Strategy 2: IHC, MS-MLPA, sequencing; Strategy 3: Revised Bethesda, IHC, MS-MLPA, sequencing

The one-way sensitivity analysis results are shown in the tornado diagrams (S1–S3 Figs in S1 File). In all the three strategies the variables that most influenced model outcomes were the number of FDRs per CRC patient, the probability of CRC (first and second) in LS carriers, the distribution of MLH1, and the distribution of MMR gene pathogenic variants.

**Fig 3:**
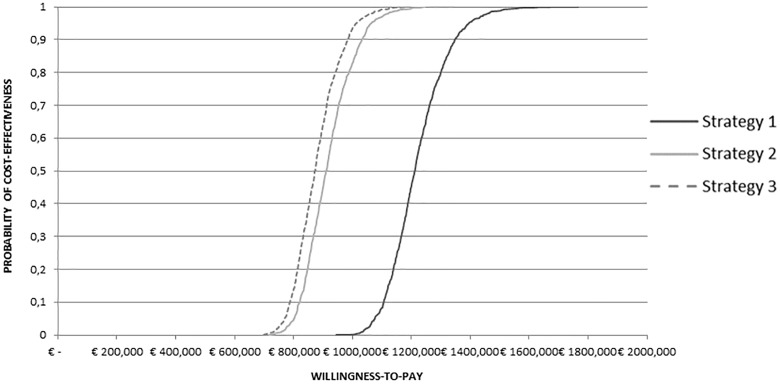
Cost-effectiveness acceptability curve.

Fig 3 shows the cost-effectiveness acceptability curves for Strategies 1–3 compared to the “No Screening strategy”. Considering a threshold of €30,000/QALY, Strategies 1,2,3 had all a 100% probability of being cost-effective as compared to “No Screening”. Moreover, Fig 3 shows, from an indirect comparison, that Strategy 3 provides the best cost-effectiveness profile; such Strategy is slightly more cost-effective than Strategy 2, and both these Strategies are significantly more cost-effective as compared to Strategy 1.

The scenario analysis revealed that removing aspirin prophylaxis from the preventive interventions has only a small influence on the cost-effectiveness of LS screening. If aspirin is omitted from the model, the ICERs of strategy 3 is equal to €806/QALY.

## Discussion

We developed a decision model to evaluate the cost effectiveness of three LS screening programs, taking into account the perspective of the Italian National Health System.

Three CRC-based LS diagnostic strategies were evaluated: the sequencing of all MMR genes without prior tumor analysis (Strategy 1), a sequential IHC and MS-MLPA analysis (Strategy 2), and an age-targeted strategy with a revised Bethesda criteria assessment before IHC and MS-MLPA for patients ≥ than 70 years old (Strategy 3). The diagnostic strategies were selected taking into account Italian expert opinions and the international state of the art [10, 19, 23].

It has been established that the selection of patients for genetic testing in LS can be improved combining IHC with MS-MLPA. This strategy is more cost-effective than the most currently used strategy (BRAF V600E mutation [44]) and requires similar technology, generally available in molecular genetic facilities [27]. Other studies analyzed age-targeted CRC-based screening programs showing that an age cutoff of 70 years is better than a lower age cutoff in terms of cost-effectiveness [23].

As already reported in Grosse and Snowsill et al. [22, 42], our study shows that universal LS screening (Strategy 1) is cost-effective compared with not performing genetic screening (No screening), but not necessarily compared with age-targeted testing strategies (Strategy 3). The most cost-effective strategy, indeed, is Strategy 3, with an ICER of €941.24/per QALY as compared to the “No Screening” strategy.

Genomic profiling has the potential to improve the delivery of patient-centered care. Still, there is a considerable gap between the discoveries in the research field and the translation of such findings into genetic services that can ultimately benefit the patients and their families.

The importance of LS identification is a priority topic, as stressed also by the US Department of Health and Human Services that adopted the initiative known as Healthy People 2020, with one of the developmental objective being: “Increase the proportion of persons with newly diagnosed colorectal cancer who receive genetic testing to identify Lynch syndrome (or familial colorectal cancer syndromes)”. Genetics in healthcare is developing rapidly and the responsibilities of genetic and non-genetic specialists are changing, requiring genetic knowledge and skills among non-genetic health care providers as well as a close collaboration and communication among them. Hence, the implementation of guidelines suggesting universal screening of all CRC patients requires a multidisciplinary approach that might not be easily achievable in all clinical contexts. Indeed, both the practice and current recommendations about LS syndrome screening are highly heterogeneous across EU Member states, and LS screening is not performed in every European country.

To date, no organized screening pathways are in place in Italy to identify LS carriers. Moreover, genetic services are not always established as part of diagnostic and therapeutic pathways in all the hospital settings [18].

In this framework, the Italian Ministry of Health is strengthening the efforts to implement adequate diagnostic programs for the management of the highly penetrant hereditary forms of cancers within both the *National Prevention Plan 2014–2018* and the *National plan for the innovation of the health system* based on the omic sciences [21, 45].

The design of the screening program and the implementation process have to be tailored to the characteristics of the health-care system. The present economic evaluation, together with the semi-structured interviews with Italian experts [19], may support decision-makers by providing important insights on how to allocate resources to new genetic screening approaches in an efficient and equitable manner, in order to ensure the translation of cost-effectiveness evidence into real-world.

The comparison with other studies is complex because diagnostic strategies showed a high variability, and parameters and assumptions included in the economic models differed according to the specific features of the screening programs evaluated [23]. As done in previous studies [24, 35], we also analyzed the effect of aspirin chemoprevention in LS carriers, but, as reported by Severin, we found that aspirin prophylaxis has only a small influence on the cost-effectiveness of LS screening, in agreement with previous findings [24]. Deterministic sensitivity analysis showed that the number of FDRs tested per patient is a critical factor for cost-effectiveness, and therefore further research should investigate factors that could improve FDR's willingness to undergo the test.

The strengths of our analysis include the adoption of promoter methylation testing, the consideration of multiple clinical and tumor-based strategies, and the incorporation of endometrial cancer risk. Moreover, we have calculated outcomes in terms of quality-adjusted life-years.

On the other hand, our analysis has several limitations. First, we did not model the risk of other cancers (ovarian, stomach.) because the benefits of screening for these type of cancer is uncertain and we did not consider different CRC risks based on the MMR genes involved. Recent studies reported that lifetime risk of developing CRC is highest for MLH1 and MSH2 mutation carriers, and CRC develops less frequently and at a later age in individuals with a mutation in MSH6 or PMS2 [46]. However, to date in Italy all LS carriers are currently offered the same surveillance interval regardless of the gene involved. Lastly, our analysis considered only direct medical costs associated to the screening.

## Conclusions

Our study evaluated the cost-effectiveness of LS screening in Italy. The results show that undertaking any LS screening is always cost-effective as compared to the “no screening” strategy based on the evidence that it would be totally sustainable by the Italian National Health Service. In particular, Strategy 3 (an age-targeted strategy with a revised Bethesda criteria assessment before IHC and MS-MLPA for patients ≥ 70 years old) is the most cost-effective Strategy compared to Strategy 1 (sequencing of all MMR genes without prior tumor analysis) and Strategy 2 (sequential IHC and MS-MLPA analysis).

## Supporting information

S1 File (DOC)
