# Supplementary material for: Cost-effectiveness analysis of genetic diagnostic strategies for Lynch syndrome in Italy
Source: PLoS One. 2020 Jul 1;15(7):e0235038. doi: 10.1371/journal.pone.0235038 (PMC7329085; doi:10.1371/journal.pone.0235038)
Supplement: S1 File — (DOC) [file pone.0235038.s001.doc]

**S1 Table: Main model parameters**

| **Parameter** | **Base case value** | **SE** | **Source** |
| --- | --- | --- | --- |
| **Population characteristics** | | | |
| CRC incidence rate | 0.09% | - | [1] |
| Prevalence of LS among CRC patients | 0.0281 | 0.000105 | [2, 3] |
| Number of FDR per CRC patient | 3.833 | 0.564 | [3] |
| Proportion of relatives with mutation | 0.44 | - | [4] |
| Acceptance of prophylactic surgery from women with LS | 0.19 |  | [5] |
| Age on entry (relatives with LS) | 43.2 |  | [4] |
| Distribution of MMR mutations: |  |  | [6] |
| *MSH2* | 0.384 | 0.144 |  |
| *MLH1* | 0.324 | 0.121 |  |
| *MSH6* | 0.144 | 0.054 |  |
| *PMS2* | 0.148 | 0.055 |  |
| **Diagnostic parameters** | | | |
| *IHC test* | | | |
| sensitivity | 0.87 | 0.00235 | [6] |
| specificity | 0.910 | 0.000157 | [6] |
| *Methylation analysis of MLH1 by MS-MLPA* |  |  |  |
| sensitivity | 0.893 | 0.0022 | [6] |
| specificity | 0.9 | 0.0000754 | [6] |
| *Direct sequencing of all MMR genes* | | | |
| sensitivity | 0.995 | 0.00289 | [6] |
| specificity | 1.000 | 0.000173 | [6] |
| *Revised Bethesda criteria* | | | |
| sensitivity | 0.881 | 0.00133 | [7] |
| specificity | 0.544 | 0.367 | [7] |
| **Disease epidemiology** | | | |
| Probability CRC for LS carriers | 0.009 | 0.0033 | [8] |
| *Stage distribution (for first and second CRC diagnosis)* |  |  |  |
| *LS carriers with biennal colonoscopy* |  |  | [9] |
| Stage 1 | 0.694 | 0.0126 |  |
| Stage 2 | 0.25 | 0.0119 |  |
| Stage 3 | 0.0556 | 0.00628 |  |
| Stage 4 | 0.0004 | - |  |
| *LS carriers NO colonoscopy* |  |  | [9] |
| Stage 1 | 0.267 | 0.0285 |  |
| Stage 2 | 0.533 | 0.0322 |  |
| Stage 3 | 0.133 | 0.0219 |  |
| Stage 4 | 0.067 | 0.0161 |  |
| Probability second CRC cancer for LS carriers | 0.0173 | 0.00648 | [10] |
| *Probability of death of patients with CRC by stage and  years from diagnosis* |  |  | [8] |
| stage 1, 1 year | 0.03 |  |  |
| stage 1, 2 year | 0.03 |  |  |
| stage 1, 3 year | 0.04 |  |  |
| stage 1, 4 year | 0.05 |  |  |
| stage 1, 5 year | 0.07 |  |  |
|  |  |  |  |
| stage 2, 1 year | 0.08 |  |  |
| stage 2, 2 year | 0.13 |  |  |
| stage 2, 3 year | 0.17 |  |  |
| stage 2, 4 year | 0.2 |  |  |
| stage 2, 5 year | 0.23 |  |  |
|  |  |  |  |
| stage 3, 1 year | 0.18 |  |  |
| stage 3, 2 year | 0.32 |  |  |
| stage 3, 3 year | 0.42 |  |  |
| stage 3, 4 year | 0.48 |  |  |
| stage 3, 5 year | 0.52 |  |  |
|  |  |  |  |
| stage 4, 1 year | 0.62 |  |  |
| stage 4, 2 year | 0.81 |  |  |
| stage 4, 3 year | 0.89 |  |  |
| stage 4, 4 year | 0.92 |  |  |
| stage 4, 5 year | 0.93 |  |  |
| Probability EC for LS carriers | 0.008 |  | [11] |
| *EC stage at diagnosis general population* |  |  | [12] |
| Stage 1 | 0.73 |  |  |
| Stage 2 | 0.09 |  |  |
| Stage 3 | 0.09 |  |  |
| Stage 4 | 0.09 |  |  |

**S2** Table: Costs

| **Genetic testing** |  |
| --- | --- |
| Immunoistochemistry | 60.00 € |
| Direct sequencing of the 4 MMR genes | 250,00 € |
| MS-MLPA | 150.00 € |
| Revised Bethesda analysis | 20.60€ |
| Sequencing one gene | 150.00€ |
| Targeted DNA testing for relative | 25.00€ |
| **Prevention in LS carriers** |  |
| Colonoscopy | 86.80€ |
| Aspirin treatment (100mg/die) for one month | 1.41€ |
| Gynecologic visit | 20.66€ |
| Transvaginal ultrasonography | 43.38€ |
| Endometrial sampling | 14.10€ |
| Prophylactic total abdominal hysterectomy | 3027.00€ |
| **Complications of prevention** |  |
| Cost of hospital treatment of complications bleeding after colonoscopy | 3,619€ |
| Cost of hospital treatment of complications perforation after colonoscopy | 19,790€ |
| Cost of hospital treatment of gastrointestinal bleeding after aspirin assumption | 2000€ |
| **CRC treatment cost** |  |
| Stage 1 | 10,126.20€ |
| Stage 2 | 11,425.40€ |
| Stage 3 | 13,374.20€ |
| Stage 4 | 28,535.10€ |
| **EC treatment cost** |  |
| Stage 1 | 5336.00€ |
| Stage 2 | 8549.00€ |
| Stage 3 | 11,090.00€ |
| Stage 4 | 2541.00€ |
| **Annual Surveillance post CRC** | 769.63€ |
| **Annual Surveillance post EC** | 363.16€ |

**Figure S1**: One-Way sensitivity analyses for Strategy 1 “Next Generation Sequencing” versus the no screening strategy.

**
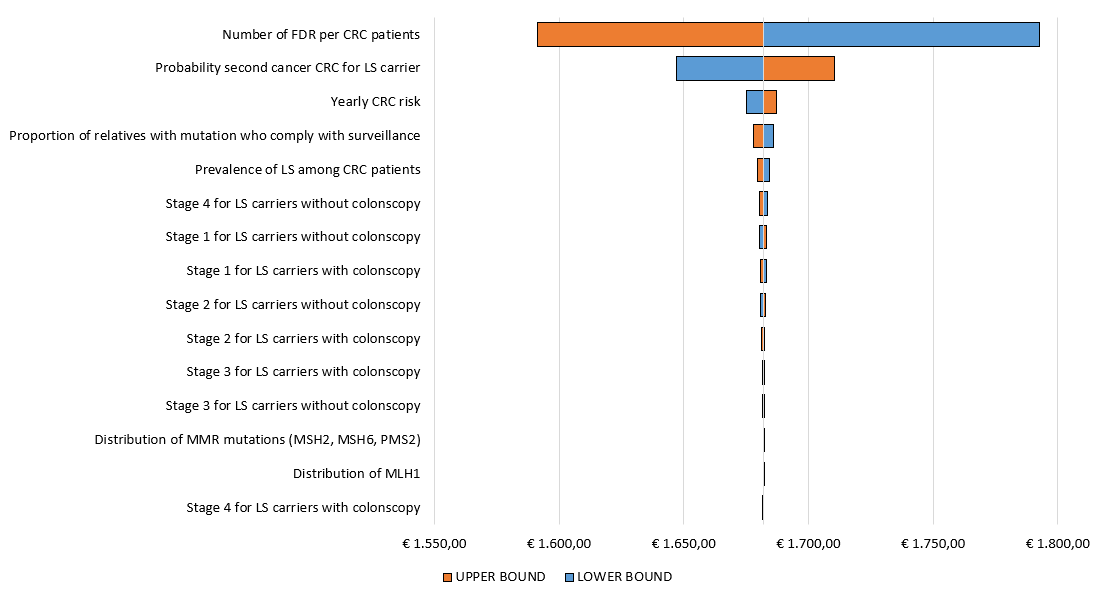
**

**Figure S2**: One-Way sensitivity analyses for Strategy 2 “IHC, MS-MLPA, sequencing” versus the

no screening strategy.

**
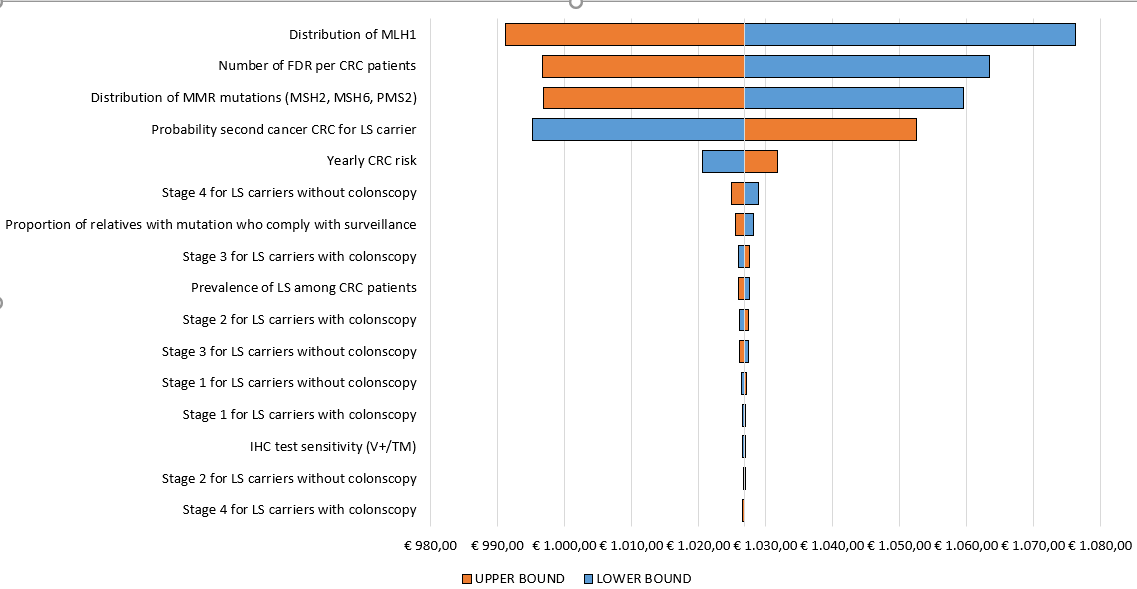
**

**Figure S3**: One-Way sensitivity analyses for Strategy 3 “Revised Bethesda, IHC, MS-MLPA, sequencing” versus the no screening strategy.

**
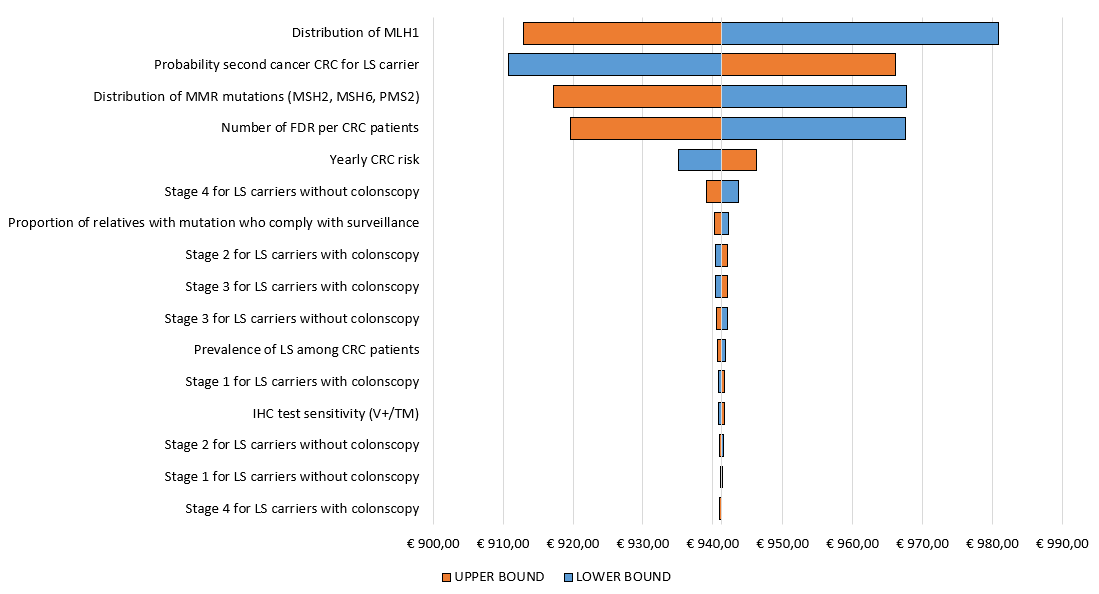
**

**Table References**

[1] I numeri del cancro in Italia 2019 - AIOM, https://www.aiom.it/i-numeri-del-cancro-in-italia/ (accessed 14 April 2020).

[2] Hampel H, Frankel WL, Martin E, et al. Feasibility of Screening for Lynch Syndrome Among Patients With Colorectal Cancer. *J Clin Oncol* 2008; 26: 5783–5788.

[3] Severin F, Stollenwerk B, Holinski-Feder E, et al. Economic evaluation of genetic screening for Lynch syndrome in Germany. *Genet Med* 2015; 17: 765–773.

[4] Snowsill T, Coelho H, Huxley N, et al. Molecular testing for Lynch syndrome in people with colorectal cancer: systematic reviews and economic evaluation. *Health Technol Assess (Rockv)* 2017; 21: 1–238.

[5] Schmeler KM, Lynch HT, Chen L, et al. Prophylactic surgery to reduce the risk of gynecologic cancers in the Lynch syndrome. *N Engl J Med* 2006; 354: 261–9.

[6] Palomaki GE, McClain MR, Melillo S, et al. EGAPP supplementary evidence review: DNA testing strategies aimed at reducing morbidity and mortality from Lynch syndrome. *Genet Med* 2009; 11: 42–65.

[7] Moreira L, Balaguer F, Lindor N, et al. Identification of Lynch Syndrome Among Patients With Colorectal Cancer. *JAMA* 2012; 308: 1555.

[8] Snowsill T, Huxley N, Hoyle M, et al. A systematic review and economic evaluation of diagnostic strategies for Lynch syndrome. *Health Technol Assess (Rockv)* 2014; 18: 1–406.

[9] Engel C, Rahner N, Schulmann K, et al. Efficacy of Annual Colonoscopic Surveillance in Individuals With Hereditary Nonpolyposis Colorectal Cancer. *Clin Gastroenterol Hepatol* 2010; 8: 174–182.

[10] Parry S, Win AK, Parry B, et al. Metachronous colorectal cancer risk for mismatch repair gene mutation carriers: the advantage of more extensive colon surgery. *Gut* 2011; 60: 950–7.

[11] Bonadona V, Bonaïti B, Olschwang S, et al. Cancer risks associated with germline mutations in MLH1, MSH2, and MSH6 genes in Lynch syndrome. *JAMA* 2011; 305: 2304–10.

[12] Havrilesky LJ, Maxwell GL, Myers ER. Cost-effectiveness analysis of annual screening strategies for endometrial cancer. *Am J Obstet Gynecol* 2009; 200: 640.e1-640.e8.
